# Modulation of Tregs and iNKT by Fingolimod in Multiple Sclerosis Patients

**DOI:** 10.3390/cells10123324

**Published:** 2021-11-26

**Authors:** Diana Ferraro, Sara De Biasi, Anna Maria Simone, Riccardo Orlandi, Milena Nasi, Francesca Vitetta, Marcello Pinti, Marco Fogliani, Stefano Meletti, Andrea Cossarizza, Patrizia Sola

**Affiliations:** 1 Department of Biomedical, Metabolic and Neurosciences, University of Modena and Reggio Emilia, 41126 Modena, Italy; 88552@studenti.unimore.it (M.F.); stefano.meletti@unimore.it (S.M.); 2 Neurology Unit, Azienda Ospedaliero-Universitaria of Modena, 41126 Modena, Italy; vitetta.francesca@aou.mo.it (F.V.); sola.patrizia@aou.mo.it (P.S.); 3 Department of Medical and Surgical Sciences for Children and Adults, University of Modena and Reggio Emilia, 41125 Modena, Italy; sara.debiasi@unimore.it (S.D.B.); andrea.cossarizza@unimore.it (A.C.); 4 Neurology Unit, Ramazzini Hospital, 41012 Carpi, Italy; annamariasimone@gmail.com; 5 Department of Neurosciences, Biomedicine and Movement Sciences, University of Verona, 37129 Verona, Italy; riccardo.orlandi@univr.it; 6 Department of Surgery, Medicine, Dentistry and Morphological Sciences, University of Modena and Reggio Emilia, 41125 Modena, Italy; milena.nasi@unimore.it; 7 Department of Life Sciences, University of Modena and Reggio Emilia, 41125 Modena, Italy; marcello.pinti@unimore.it

**Keywords:** multiple sclerosis, fingolimod, T regulatory cells, iNKT cells

## Abstract

The altered numbers and functions of cells belonging to immunoregulatory cell networks such as T regulatory (Tregs) and invariant Natural Killer T (iNKT) cells have been reported in Multiple Sclerosis (MS), an immune-mediated disease. We aimed to assess the frequencies of Tregs and iNKT cells in MS patients throughout a one-year treatment with fingolimod (FTY) and to correlate immunological data with efficacy and safety data. The percentage of Tregs (defined as Live Dead-CD3 + CD4 + FoxP3 + CD25++/CD127− cells) increased steadily throughout the year, while there was no significant difference in the absolute number or percentage of iNKT cells (defined as CD3 + CD14−CD19− Vα24-Jα18 TCR+ cells). However, out of all the iNKT cells, the CD8+ iNKT and CD4−CD8− double-negative (DN) cell percentages steadily increased, while the CD4+ iNKT cell percentages decreased significantly. The mean percentage of CD8+ T cells at all time-points was lower in patients with infections throughout the study. The numbers and percentages of DN iNKT cells were more elevated, considering all time-points, in patients who presented a clinical relapse. FTY may, therefore, exert its beneficial effect in MS patients through various mechanisms, including the increase in Tregs and in iNKT subsets with immunomodulatory potential such as CD8+ iNKT cells. The occurrence of infections was associated with lower mean CD8+ cell counts during treatment with FTY.

## 1. Introduction

Multiple Sclerosis (MS) is considered an immune-mediated disease, characterized by the activation of lymphocytes directed against central nervous system autoantigens. The altered numbers and functions of cells belonging to immunoregulatory cell networks such as T regulatory (Tregs) and invariant Natural Killer T (iNKT) cells have been reported in MS [1,2,3,4,5,6,7,8,9].

Tregs play a role in autoimmunity and supposedly control autoreactivity. Numerous studies have shown altered numbers or functions of FoxP3 Tregs, both in the animal model of MS (EAE) [1,2] and in patients [4,10]. Disease-modifying drugs (DMD) such as beta-interferon and glatiramer acetate in MS may even, in part, exert their beneficial effect by increasing the number or functionality of Tregs [11,12,13].

Invariant Natural Killer T cells (iNKT) are CD1d-restricted T cells characterized by the expression of an invariant TCR alpha-chain (Vα24-Jα18) and markers of NK cells. They are potent cytokine producers, have immunoregulatory potential and can be divided into functionally distinct subsets on the basis of the expression of CD4 and CD8. They are implicated in the induction of T cell tolerance, in the prevention of autoimmune diseases, and in antimicrobial and antitumor immunity [14]. Reduced numbers of iNKT or anergy of iNKT cells have been described in MS patients [5,6,7], with a significant increase following a treatment with beta-interferon, suggesting a protective role of iNKT cells in MS [15].

Fingolimod (FTY), a superantagonist for the sphingosine-1-phosphate receptor 1 on lymphocytes, induces lymphocyte retention in lymph nodes. It predominantly affects naive and T central memory cells, which express CCR7 and CD62 ligand (CD62L) and tend to recirculate through lymphnodes on a regular basis. FTY was shown to increase the frequencies of Tregs in the peripheral blood of MS patients [16], while, to our knowledge, there is no data on its effect on iNKT cells.

The aim of our study was to evaluate the absolute numbers and percentages of peripheral Tregs and iNKT cells in patients with relapsing–remitting MS at baseline; after 2–3, 7, 14 and 28 days; and after 6 and 12 months of treatment with FTY. The secondary outcome of the study was to correlate immunological data with efficacy (i.e., occurrence of relapses) and safety (i.e., infections) of treatment with FTY.

## 2. Materials and Methods

### 2.1. Patients’ Selection

Patients commence FTY (0.5 mg once a day, orally) as a first- or second-line treatment with the following inclusion criteria were prospectively enrolled, with prior informed consent: (a) age between 18 and 65 years, (b) diagnosis of relapsing–remitting MS in accordance with the 2010 McDonald criteria [17] and (c) ability to sign an informed consent. The study was approved by the local ethics committee (Comitato Etico Provinciale di Modena, protocol number 1676).

At the time of enrolment, the following data was collected: patient disability as per the Expanded Disability Severity Score (EDSS), the number of relapses in the preceding year, the previous treatment, the duration of the wash-out between the previous treatment and FTY.

Patients underwent neurological assessments at baseline and at 6 and 12 months, and in case of a relapse. During each visit, data on EDSS, relapses, reasons for drug discontinuation, serious or adverse events, Magnetic Resonance Imaging (MRI) activity (presence of new or gadolinium-enhancing lesions on MRIs carried out as per clinical practice) and occurring infections (number and type) were collected.

### 2.2. Flow Cytometry

Peripheral blood (up to 20 mL per patient) was collected at each specified time-point. First, CD3+ T cells were volumetrically counted by using a CyFlow Counter (Partec) by a standardized no-lyse no-wash method on 100 μL of whole blood [18]. Second, PBMCs were isolated on Ficoll according to standard procedures [9] and stained with different mAbs using the following directly conjugated mAbs: anti-TCR Vα24-Jα18 PE, anti-CD4 APC-H7, anti-CD8 AF647, anti-CD161 FITC, anti-CD3 PE-Cy7 (from Becton Dickinson, San José, CA, USA), anti-CD19 ECD and anti-CD14 ECD mAbs (from Beckman Coulter, Miami, FL, USA). Cells were incubated for 20 min at room temperature, washed and acquired immediately.

To identify Treg cells, the following mAbs were used: anti-CD3 PB, -CD4 APC-H7, -CD25 PE, -CD127 APC, -FoxP3 FITC (Becton Dickinson) and LIVE/DEAD Red Fixable (ThermoFisher, Eugene, OR, USA). Cells were incubated 20 min in RT and washed. Cells were washed, fixed and permeabilized using a Human FoxP3 Buffer Set (Becton Dickinson). Cells were incubated with anti-FoxP3 FITC. Cells were washed and immediately acquired by using an Attune NxT flow cytometer equipped with four lasers (405, 488, 561, and 643 nm). Data were analyzed with FlowJo 9.9.6 (Becton Dickinson) under MacOS X. The gating strategies used for the identification of Tregs and iNKT cells are reported in Supplementary Figures (Figures S1 and S2, respectively).

### 2.3. Statistics

The Mann–Whitney test was used for comparing the continuous variables between groups and the chi-square test for categorical variables. The Bonferroni correction was applied for multiple testing. The Skillings–Mack test, followed by a post hoc Kruskal–Wallis analysis was used for longitudinal data analysis. The data were analyzed using STATA 11 (StataCorp, College Station, TX, USA).

## 3. Results

### 3.1. Patients

We enrolled 36 patients in the study. Ten patients discontinued the drug: three due to relapses (one of which lead to hospitalization—a serious adverse event), one due to recurrent primary varicella, one due to herpes zoster, two due to the elevation of liver function tests, one due to lymphopenia and two due to their own choice. Two patients dropped out of the study (moved to a different city and a different MS center). Cytoflurimetric, clinical and MRI data were available for 28 patients at least until the six-month visit. Complete clinical, MRI and cytofluorimetric data were available for 20 patients at the 12 months visit. Table 1 shows patients’ baseline and follow-up clinical and MRI data. Ten patients presented a relapse, and seven patients presented infections (four urinary tract infections, one otitis, one influenza and two VZV-related infections). The mean wash-out duration was 4 weeks following the beta-interferon or glatiramer acetate and 16 weeks following the natalizumab discontinuation.

### 3.2. Cytofluorimetric Analysis

#### 3.2.1. Longitudinal Analysis: CD3+, CD4+, CD8+ and Tregs

**Figure 1:**
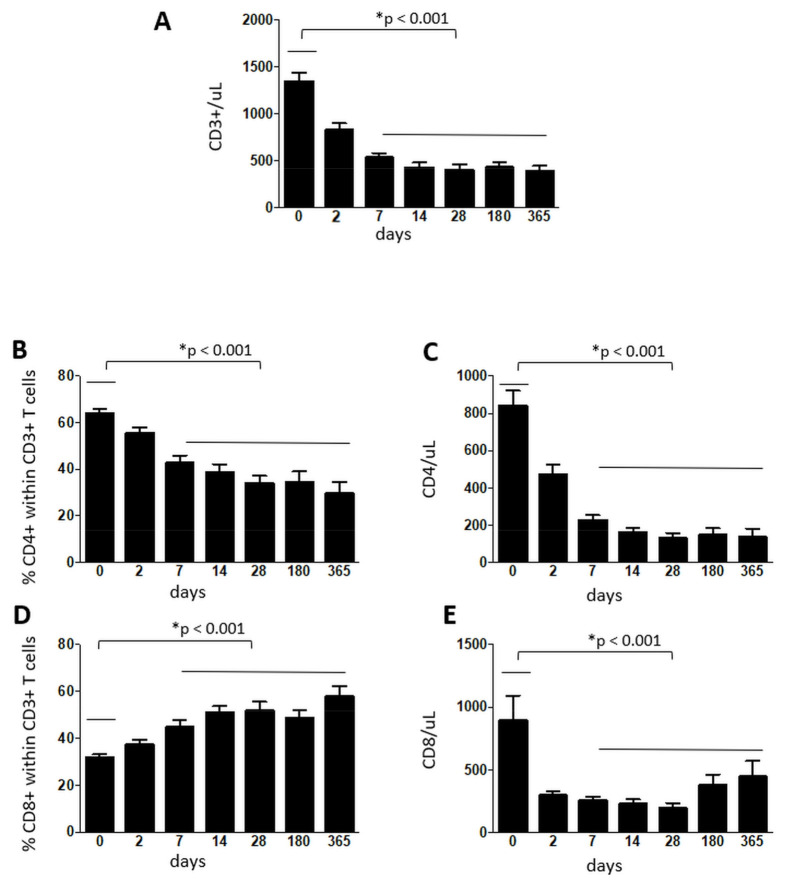
Absolute numbers of CD3+ cells (panel **A**), percentage (panel **B**) and absolute numbers (panel **C**) of CD4+ cells among CD3+ cells and percentage (panel **D**) and absolute numbers (panel **E**) of CD8+ cells among CD3+ cells derived from MS patients before starting fingolimod (0) and at different time-points throughout one year. Data are represented as mean ± standard error of the mean. *N* = 36 patients throughout days 0–28, *N* = 28 at 180 days and *N* = 20 at 365 days.

**Figure 2:**
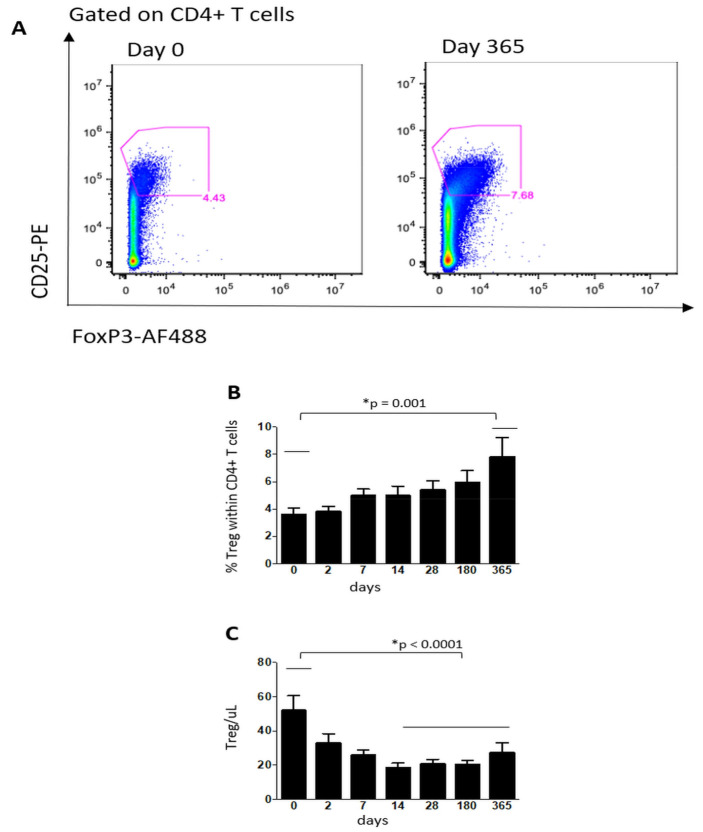
Representative dot plots showing the percentages of Treg cells for the same patients before and after 1 year of therapy (panel **A**). Percentage among CD4+ cells (panel **B**) and absolute numbers (panel **C**) of Tregs derived from MS patients before starting fingolimod (0) and at different time-points throughout one year. Data are represented as mean ± standard error of the mean. *N* = 36 patients throughout days 0–28, *N* = 28 at 180 days and *N* = 20 at 365 days.

As shown in Figure 1, the absolute numbers of CD3+, CD4+ and CD8+ T cells decreased significantly from day seven onwards, compared to the baseline (*p* < 0.001) and the percentage of CD4+ T lymphocytes decreased (*p* < 0.001), while the percentage of CD8+ T lymphocytes increased (*p* < 0.001) from day seven onwards (Figure 1). The percentage of Tregs (defined as Live Dead-CD3 + CD4 + FoxP3 + CD25++/CD127− cells) increased steadily throughout the year, with a statistical significance between the baseline and 12 months (*p* = 0.001), while the absolute number decreased significantly from day 14 onwards (*p* < 0001) (Figure 2).

#### 3.2.2. Longitudinal Analysis: iNKT Cells

**Figure 3:**
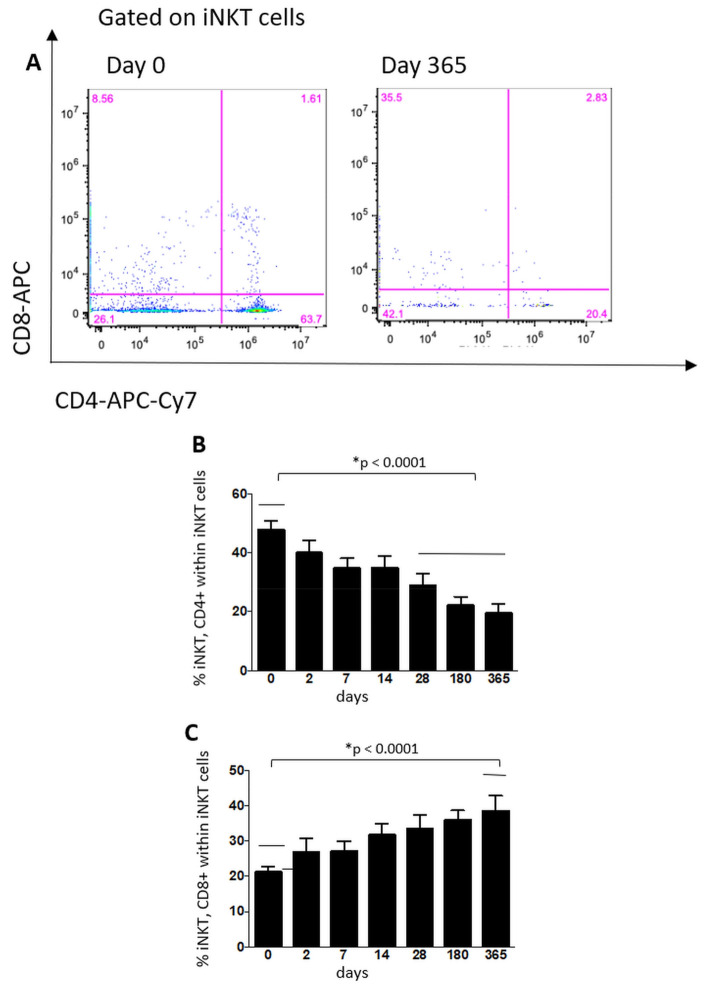
Representative dot plot showing the percentages of CD4+, CD8+ and DN iNKT cells of the same patients before and after 1 year of therapy (panel **A**). Percentage among iNKTcells of iNKT CD4+ cells (panel **B**) and iNKT CD8+ cells (panel **C**) derived from MS patients before starting fingolimod (0) and at different time-points throughout one year. Data are represented as mean ± standard error of the mean. *N* = 36 patients throughout days 0–28, *N* = 28 at 180 days and *N* = 20 at 365 days.

There was no significant difference in the absolute number or percentage of iNKT cells (defined as CD3 + CD14−CD19− Vα24-Jα18 TCR+ cells), while, out of all iNKT cells, the CD8+ iNKT cell percentages steadily increased and were significantly higher at 12 months (*p* < 0.0001) (Figure 3). Absolute values of CD8+ and CD4+ iNKT cells did not vary significantly. Out of all iNKT cells, CD4+ iNKT cells percentages decreased significantly from the 28th day onwards (*p* < 0.0001) (Figure 3). DN iNKT cell percentages significantly increased at six months (*p* < 0.001), while their absolute numbers did not vary.

#### 3.2.3. Correlations between Clinical and Cytofluorimetric Data

The following cells were compared between patients with and without infections and between patients with and without relapses throughout the study period: CD3+, CD4+, CD8+, Treg, iNKT, CD8+ iNKT, CD4+ iNKT and CD4−CD8− iNKT. Comparisons of the above-mentioned cell subsets between males and females were also carried out, and they did not show significant differences (Figures S3–S5).

##### Comparison between Patients with and without Infections

**Figure 4:**
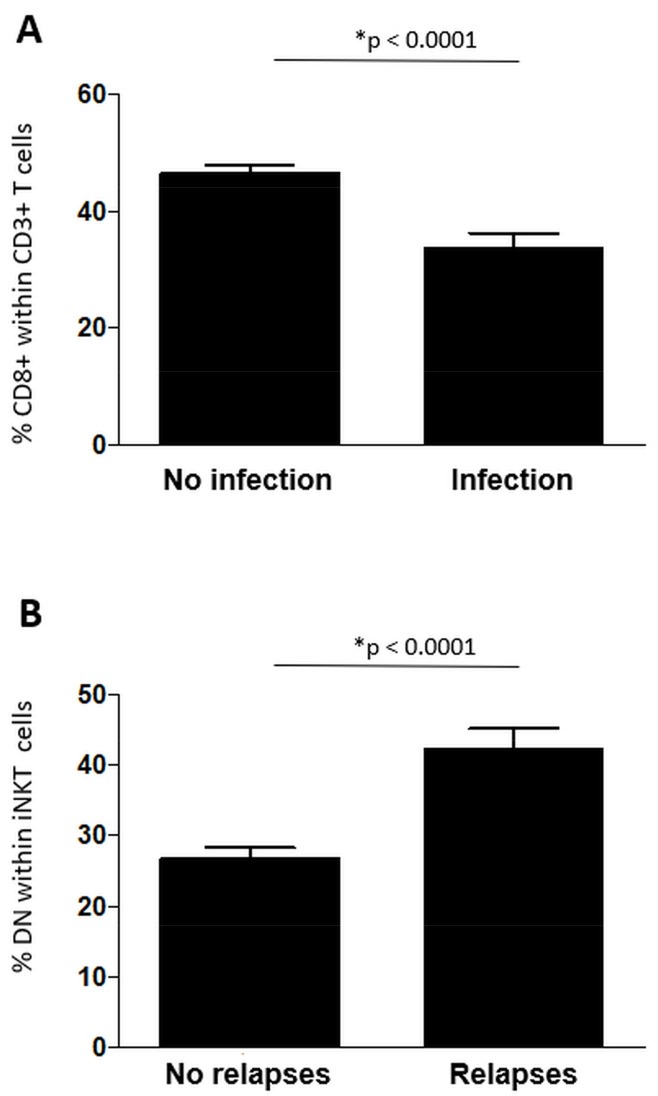
Mean percentage of CD8+ T cells in patients with (*N* = 7) and without (*N* = 29) clinically significant infections during the treatment period (panel **A**). Mean percentage of DN iNKT cells in patients with (*N* = 10) and without (*N* = 29) a clinical relapse during the treatment period (panel **B**). Data are represented as plus or minus standard error of the mean.

The mean percentage of CD8+ T cells at all time-points was lower (46.5% ± 16 versus 33.5% ± 13.8; *p* = 0.0001, significant after the Bonferroni correction) in case of an infection during treatment and, in particular, in case of a VZV infection (23.3% ± 7) (Figure 4). Patients with mean percentages of CD8+ T cells < 35% had four-fold odds of an infection (odds ratio: 4.4; 95% confidence interval: 2–10; *p* < 0.0001). The baseline CD8+ T cell percentages were, likewise, lower in patients with a subsequent infection (26.4 ± 6.5 versus 33 ± 8.7; *p* = 0.048, not significant after the Bonferroni correction). The remaining analyzed cell subsets did not differ significantly between the two groups.

##### Comparisons between Patients with and without Relapses

The mean absolute numbers and percentages (Figure 4) of DN iNKT cells were more elevated, considering all time-points, in patients who presented a clinical relapse compared to those who did not (42.3% ± 18.9 versus 26.8% ± 17 and 1548/μL ± 2424 versus 566/μL ± 1193, respectively; *p* < 0.0001, significant after the Bonferroni correction). The remaining analyzed cell subsets did not differ significantly between the two groups.

## 4. Discussion

FTY, a sphingosine 1-phosphate (S1P) receptor modulator, is an oral DMD approved for the treatment of highly active relapsing–remitting MS. With regard to the immune system, S1P is expressed on both T and B cells. FTY has been shown to selectively retain T cells that regularly traffic through lymphnodes and which express the homing receptor CCR7, including naive T cells, central memory T cells and a proportion of B cells. The binding of FTY to S1P on lymphocytes results in the internalization and degradation of the receptor. As a consequence, S1P-mediated egress of lymphocytes from lymph nodes to the periphery is blocked, thereby reducing the number of inflammatory cells migrating to the central nervous system both in animal models and in humans [19,20]. The sequestration of B and T lymphocytes in secondary lymphoid organs causes a decrease in absolute lymphocyte counts to 20–30% of the baseline values between days three and seven of treatment initiation [20]. Although the peripheral decreased lymphocyte count is a well-known phenomenon, it is unknown whether distinct lymphocyte subset patterns can assist the clinician in selecting patients that are at higher risk for infections or nonresponsiveness to FTY treatment.

We aimed to shed light on Treg and iNKT cells variations throughout a one-year treatment period with FTY and to correlate them with clinical responses to treatment and safety data, with a particular emphasis on the occurrence of infections.

As expected, the number and percentage of CD3+, CD4+ and CD8+ T cells decreased significantly within the first seven days of treatment and remained at low values throughout the study [20].

FTY increased the circulating levels of Tregs; this is consistent with other studies [16,19,21] and with the capability of restoring Treg homeostasis in MS patients [3,4,22]. Treg cells are essential for maintaining peripheral tolerance, preventing autoimmunity and limiting chronic inflammatory diseases, although they may also limit beneficial responses by suppressing the sterilizing immunity to certain pathogens [23,24]. The beneficial effect of other DMDs such as interferon-beta and glatiramer acetate have also been postulated to be mediated partly by an expansion of Tregs [11,12]. The majority of human Tregs lack expression of CCR7, which explains the present findings and why Tregs are not targeted by S1P-mediated chemotaxis [25].

Several studies have assessed iNKT cell numbers and functions in MS [26]. Some studies report a reduced frequency of iNKT [5,6,27,28], while others report increased [7] or unvaried frequencies compared to healthy controls [9,29]. This might be due to several reasons, including the selection of patients under investigation, their therapy or even the methodology used. With regard to their function, iNKT cells from patients with MS were found to be defective in cytokine production [27,29], to be hyporesponsive to stimulation with α-GalCer in vitro [7,8] and to have a Th1/Th17 cytokine bias, which was most prominent in patients with secondary progressive MS, who displayed an increased production of IL-17 by either CD4+ CD8− or CD4−CD8+ iNKT cells [30]. MS patients treated with interferon-beta showed increased percentages of iNKT cells among peripheral blood mononuclear cells and an improved cytokine secretion in response to α-GalCer [15], while patients treated with natalizumab displayed lower levels of iNKT cells producing IL-17, TNF and IFN-gamma compared to patients on interferon-beta or glatiramer acetate [9].

In the present study, the number and percentage of iNKT did not change during treatment of FTY. However, we observed that within iNKT cells, the percentage of those expressing CD4 decreased in accordance with the CD4+ T cell population, while those that were CD8+ or DN increased during treatment, and increased values of DN cells were associated with the occurrence of relapses during treatment. FTY did not alter the total numbers of circulating iNKT, probably because only a small fraction of iNKT express CCR7 [30] and, amongst these, it is mainly CD4+ CD8− iNKT cells that express greater levels of CCR7 e CD62L. Indeed, a study assessing homingreceptor expression by different human iNKT cell subsets in circulation found a small but distinct CD62L+ and CCR7+ subset within the iNKT cell population, but most of the iNKT cells were CD4+, a few were DN and virtually none were CD8+ [31]. This could explain the decrease in circulating levels of CD4+ CD8− iNKT found in the present study. The relative increase in CD4−CD8+ cytotoxic cells, potentially implicated in the suppression of activated T cell expansion [32], may be beneficial in MS patients. With regard to DN iNKT cells, they were shown to be skewed towards a Th1 phenotype in relapsing–remitting MS patients, who exhibited lower frequencies of IL-4 secreting DN iNKT cells [29]. Interestingly, in the present study, patients with relapses during FTY treatment had higher numbers and percentages of DN cells compared to those without relapses.

Patients with infectious events during the study had lower mean percentages of CD3−CD8+ cells, which is consistent with the fact that CD8+ T cells are paramount for the successful control of a vast majority of viral infections [33]. This information may be useful for risk stratification and for decisions during treatment with FTY.

Limits in the interpretation of data regarding iNKT cells must be acknowledged, since they can exert a proinflammatory function and a tolerogenic function depending on the context of the antigenic stimulation and on the integration of different costimulatory signals [14]. Furthermore, a more extensive characterization of human NKT cells from tissues and organs is needed and little is known about the distribution of NKT cell subsets in humans.

## 5. Conclusions

FTY may exert its beneficial effect in MS patients through various mechanisms, including the increase in Tregs and in iNKT subsets with immunomodulatory potential such as CD8+ iNKT cells. Its effect on other iNKT subsets, such as DN cells and the possible association between an increase in DN cells and the risk of relapses needs to be further studied. Finally, patients with infections during FTY treatment had lower mean percentages of CD8+ cells throughout the study.

## Figures and Tables

**Table 1:** Patient characteristics.

**Sex**	
Males, *n* (%)	13 (36)
Females, *n* (%)	23 (64)
**Age**	
Mean (SD)	39.3 (9.3)
Range	23–60
**Basal EDSS**	
Mean (SD)	3.3 (1.8)
Range	0–6.5
**EDSS at 6 months** (29 pts)	
Mean (SD)	3.2 (2.1)
Range	0–6.5
**EDSS at 12 months** (24 pts)	
Mean (SD)	3 (2)
Range	0–6.5
**Relapses during the study**	
0 relapses, *n* (%)	25 (71)
1 relapse, *n* (%)	10 (29)
**EDSS increase**	
Present at 6 months, *n* (%)	2 (6)
Present at 12 months, *n* (%)	4 (17)
**Number of new or gadolinium-enhancing lesions at 6 months** (29 pts)	
0 lesions, *n* (%)	18 (62)
1–3 lesions, *n* (%)	6 (21)
4–7 lesions, *n* (%)	2 (7)
8–10 lesions, *n* (%)	3 (10)
**Number of new or gadolinium-enhancing lesions at 12 months** (21 pts)	
0 lesions, *n* (%)	15 (71)
1–3 lesions, *n* (%)	5 (10)
4–7 lesions, *n* (%)	5 (5)
8–10 lesions, *n* (%)	0 (0)
**Previous treatment**	
Glatiramer acetate, *n* (%)	7 (19)
Beta-interferon 1a or b, *n* (%)	18 (50)
Natalizumab, *n* (%)	11 (31)

## Data Availability

The data presented in this study are available from the corresponding author upon request.

## Supplementary Materials

The following are available online at https://www.mdpi.com/article/10.3390/cells10123324/s1. Figure S1: Gating strategy used to identify Treg cells. Figure S2: Gating strategy used to identify iNKT cells. Figure S3: CD3+, CD4+ and CD8+ among men and women throughout the study. Figure S4: Tregs among men and women throughout the study. Figure S5: Total iNKT, CD4+ iNKT, CD8+ iNKT and DN iNKT among men and women throughout the study.
