# Supplementary figures and images for: Modulation of Tregs and iNKT by Fingolimod in Multiple Sclerosis Patients
Source: Cells. 2021 Nov 26;10(12):3324. doi: 10.3390/cells10123324 (PMC8699557; doi:10.3390/cells10123324)

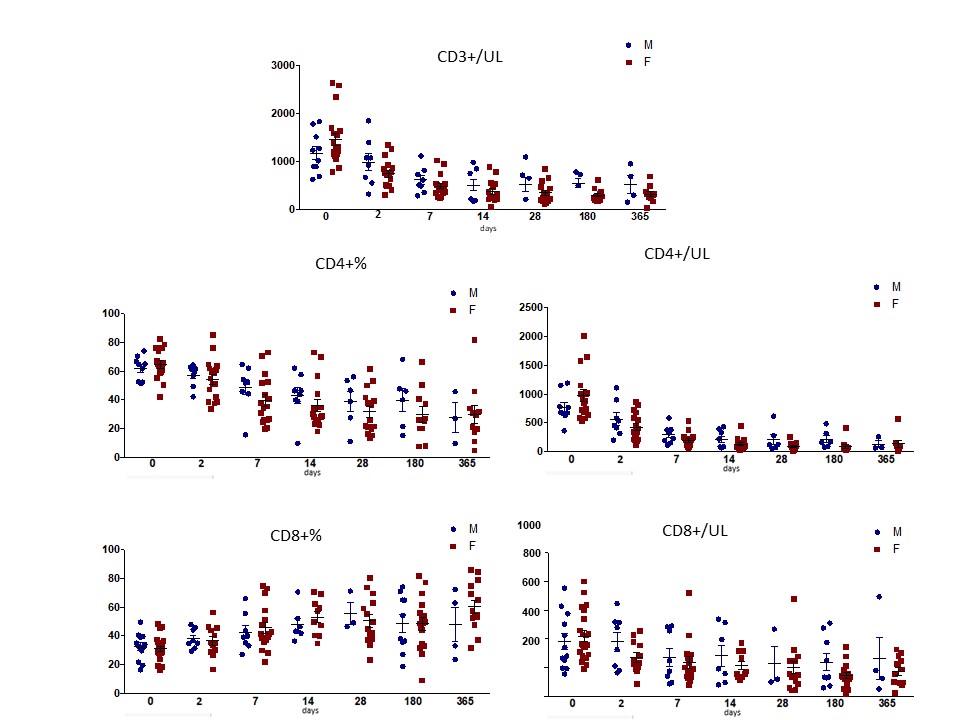

Supplement: Supplementary file 1 [file cells-10-03324-s001.zip › supplementary figure S3.JPG]

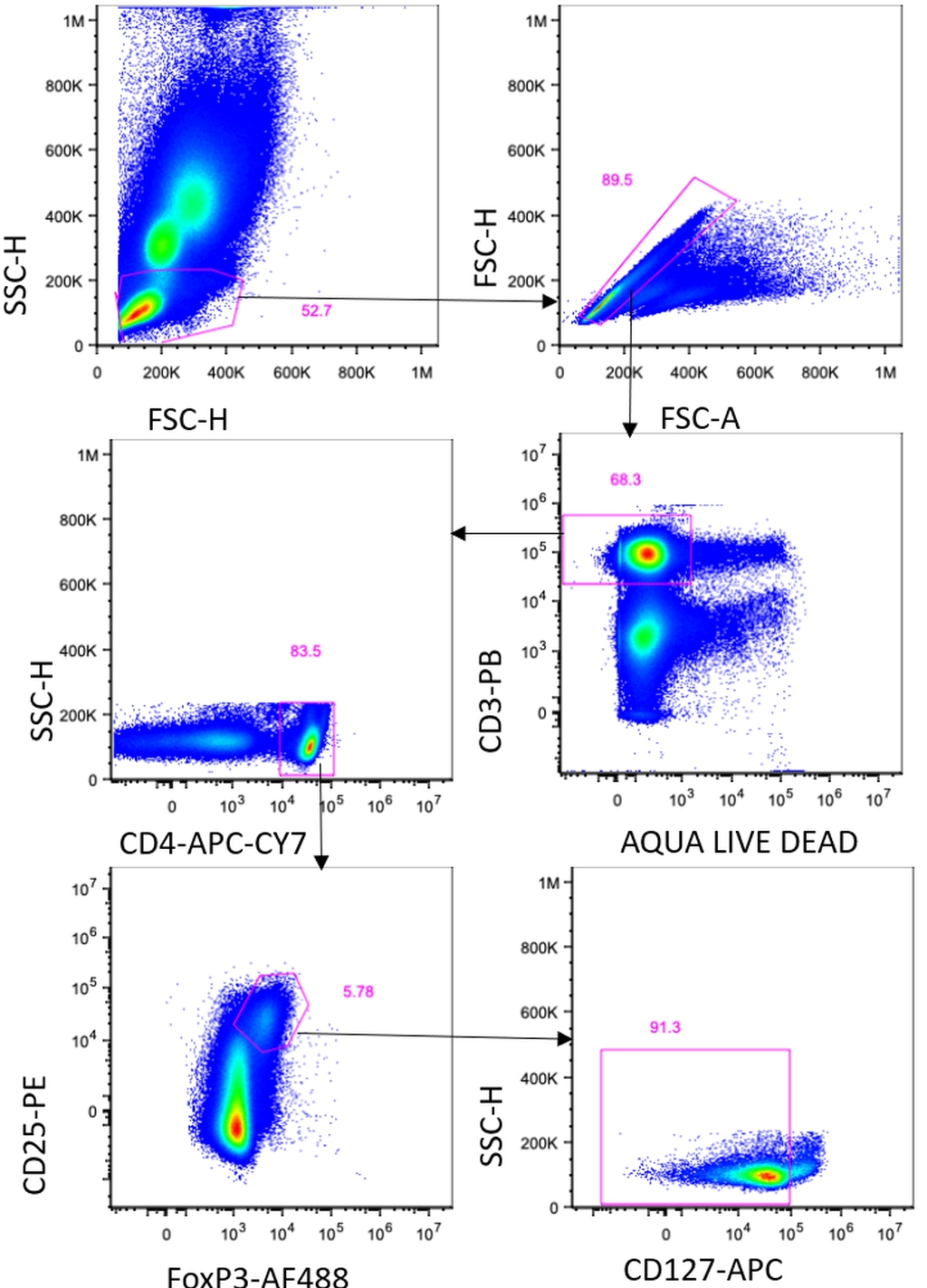

Supplement: Supplementary file 1 [file cells-10-03324-s001.zip › supplementary figure S1.jpg]

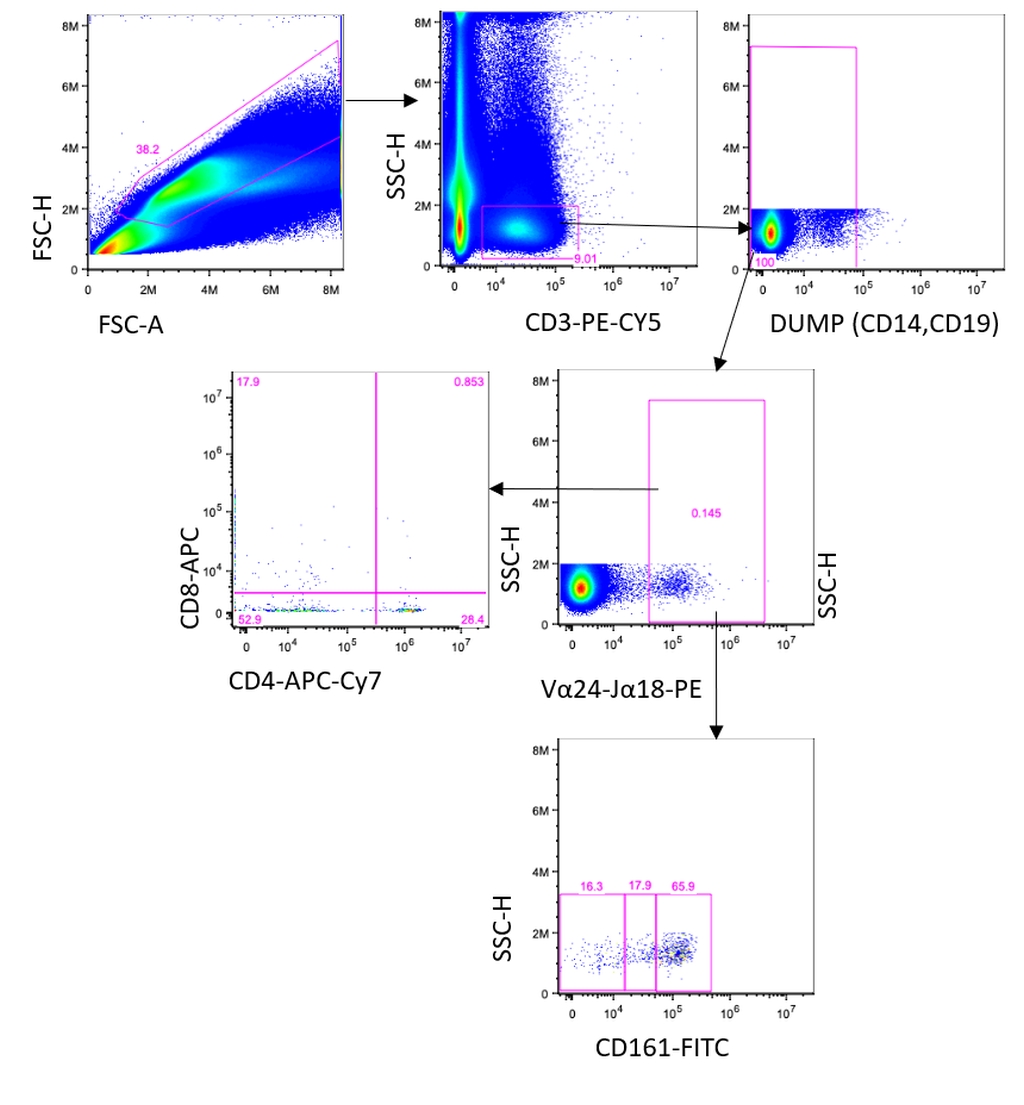

Supplement: Supplementary file 1 [file cells-10-03324-s001.zip › supplementary figure S2.jpg]

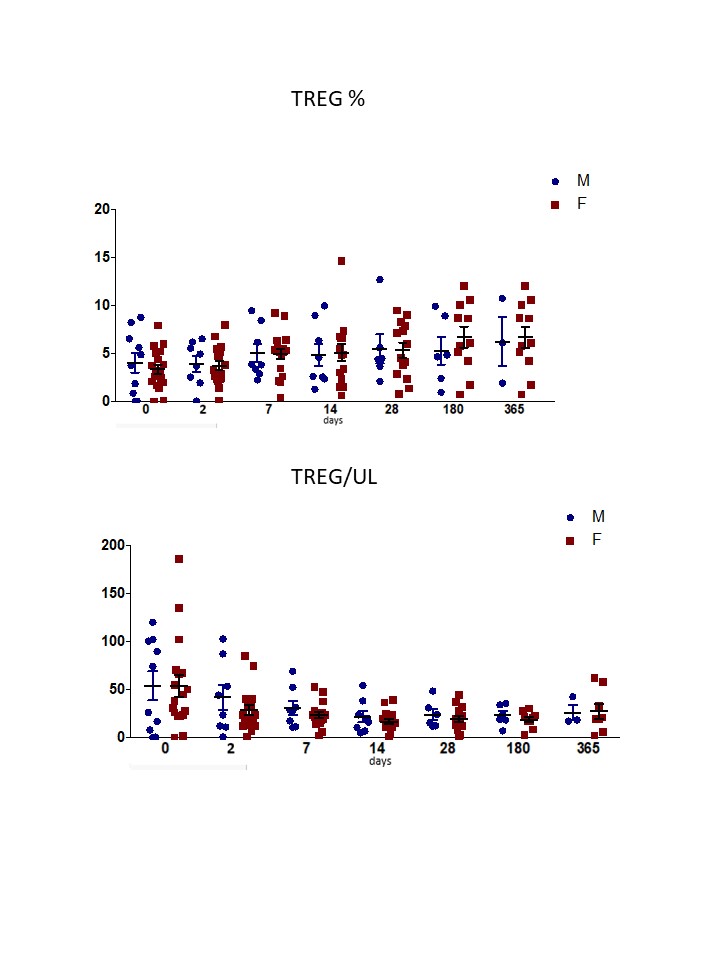

Supplement: Supplementary file 1 [file cells-10-03324-s001.zip › Supplementary figure S4.JPG]

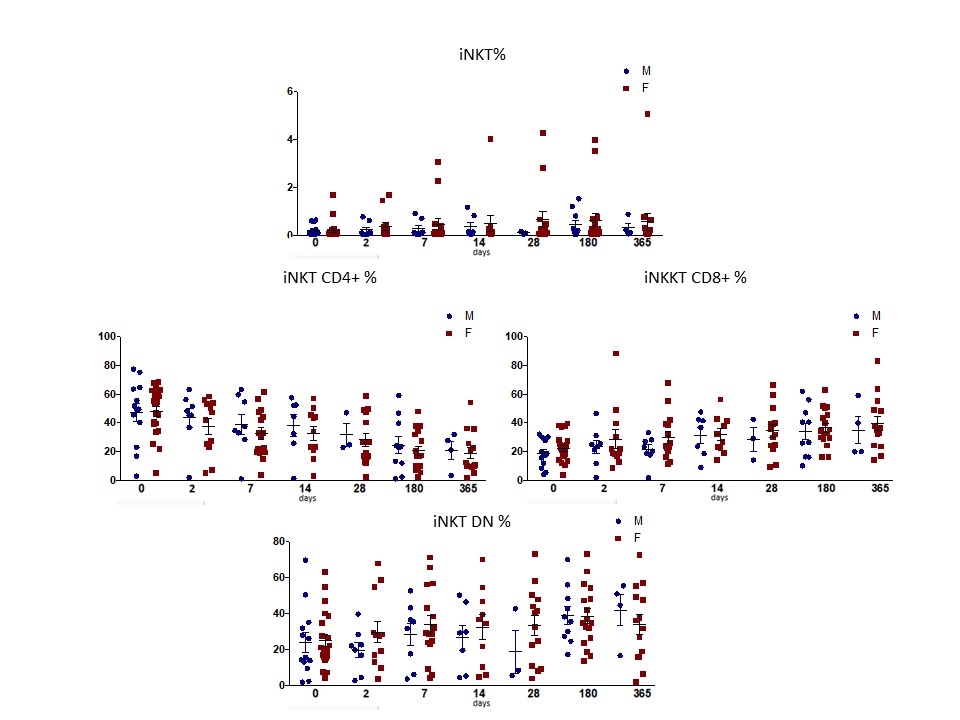

Supplement: Supplementary file 1 [file cells-10-03324-s001.zip › Supplementary figure S5.JPG]
